# Efficacy of β-Blockers on Postural Tachycardia Syndrome in Children and Adolescents: A Systematic Review and Meta-Analysis

**DOI:** 10.3389/fped.2019.00460

**Published:** 2019-11-07

**Authors:** Xinwei Deng, Yuyang Zhang, Ying Liao, Junbao Du

**Affiliations:** ^1^Department of Pediatrics, Peking University First Hospital, Beijing, China; ^2^Peking University Health Sciences Centre, Beijing, China; ^3^Research Unit of Clinical Diagnosis and Treatment of Pediatric Syncope and Cardiovascular Diseases, Chinese Academy of Medical Sciences, Beijing, China

**Keywords:** efficacy, β-blockers, metoprolol, children, POTS

## Abstract

**Background:** Postural tachycardia syndrome (POTS) is a severe health problem in children. Short-term β-blockers are recommended for pharmaceutical treatment. However, there have been contradictory data about its efficacy among pediatric patients.

**Methods and Results:** Eight studies comparing β-blockers to conventional treatments for children with POTS were selected, where 497 cases of pediatric POTS were included. The efficacy of β-blockers was evaluated using the effective rate, the change of symptom score, the change of heart rate difference and adverse events. The results were stated as relative ratio (RR) and mean difference (MD) with a 95% confidence interval (95% CI). A random-effects meta-analysis for the effective rate indicated that β-blockers were more effective in treating pediatric POTS than controlled treatment (79.5 vs. 57.3%, RR = 1.50, 95%CI: 1.15–1.96, *P* < 0.05). A fixed-effects model analysis showed that β-blockers were more effective in lowering the symptom score and the heart rate increment during standing test than controlled treatment with a mean difference of 0.81 (95% CI: 0.44–1.18, *P* < 0.05) and 3.78 (95% CI: 2.10–5.46, *P* < 0.05), respectively. There were no reported severe adverse events in included studies.

**Conclusion:** β-blockers are effective in treating POTS in children and adolescents, alleviating orthostatic intolerance, and improving hemodynamic abnormalities.

## Introduction

Postural tachycardia syndrome (POTS) is common in children, featuring an abnormal increment in heart rate of over 40 beats per minute (bpm) within the first 10 min of head-up tilt (HUT) or standing test accompanied by symptoms of orthostatic intolerance such as dizziness, headache, palpitation, chest discomfort, blurred vision, tremor, and profuse perspiration (1). The prevalence of POTS in Chinese children is ~6.8%, with a peak age of onset around 15–25 years old (2). Children are more easily affected than adults, with recurrent syncope attacks most often resulting in physical and psychological damage.

Currently used drugs for POTS include β-adrenoreceptor blockers, α-adrenoreceptor agonists, pyridostigmine and fludrocortisone, each of which has a distinct while overlapping mechanism underlying its observed clinical efficacy (3). Decreased intravascular volume, elevated plasma norepinephrine levels, attenuated sympathetic vasoconstrictor responsiveness, and peripheral autonomic neuropathy are important factors contributing to tachycardia in POTS patients (4–6). Through reducing cardiac baroreceptor activation, lowering blood norepinephrine level, and inhibiting sympathetic nerve activity, it is likely reasonable that β-blockers might be a promising therapeutic option for the treatment of POTS (7).

Although several randomized controlled trials of relatively high quality may have provided physicians with reasons to consider treating adult POTS patients with β-blockers, it is not the case with children (8, 9). Inconsistent results have been published in recent years, most of which are non-randomized, or of small sample size. A randomized controlled trial (RCT) of Lin et al. (10) in the treatment of 54 children with POTS using metoprolol showed that the treatment group was significantly more effective than the control group (72.2 vs. 48.0%), while Chen et al. (11) found that the efficacy of a same drug was unproved, also in an RCT that involved 19 POTS children. Therefore, the present study was undertaken to carry out a systematic review and meta-analysis to present updated evidence for clinical reference and hopefully to provide guidance for further research in this field.

## Methods

### Criteria for Considering Studies

#### Types of Studies

The studies included the analysis of the RCTs and non-randomized controlled trials (Non-RCTs). We only included prospected studies on the treatment of POTS in children and adolescents, comparing β-blockers to conventional therapies.

#### Types of Participants

We included pediatric patients aged below 20 years old, who were diagnosed as POTS by HUT or standing test. We excluded patients with any systematic diseases, metabolic disturbances, or cardiogenic diseases.

#### Types of Interventions

Studies that compared treatment of oral administration of β-blockers using standard pediatric doses and duration with conventional therapies were included. Conventional therapies for control group referred to non-pharmacological measures such as oral rehydration salts (ORS) and patient education. We allowed additional interventions in trials such as α-adrenergic receptor agonist if there was a comparison with β-blockers. We excluded trials with short duration of therapeutic course <1 month.

#### Types of Outcome Measures

Our primary outcome was the effective rate, a dichotomous variable defined as the ratio of participants whose symptoms were relieved after the treatment. This outcome was equal to the cure rate plus the improvement rate. Our secondary outcomes included: (1) the change of symptom score (Δ heart rate difference): defined as the reduction in symptom score according to Winker symptom scale (WSS) and expressed as mean ± standard deviation; (2) the change of heart rate difference (Δ heart rate difference): the heart rate difference is defined as the increment of heart rate during HUT, while Δ heart rate difference stands for the heart rate difference after the treatment minus the baseline heart rate difference. The results were expressed as mean ± standard deviation; (3) adverse events: defined as drug-related adverse effects. Our study documented the adverse effects reported in each trial explicitly.

### Search Strategy

We searched the following databases till 24 June 2019 without any restriction on the published years: Cochrane Library, EMBASE, Pubmed, and Sinomed. The databases were searched by two professional co-workers using search terms (in English or Chinese) such as “postural tachycardia syndrome/ postural orthostatic tachycardia syndrome/POTS” AND “treatment/therapy/intervention/management/β-blocker/metoprolol/propranol/betaloc/atenolol.” Original articles were obtained through downloads from electronic databases or copies from libraries. References of relevant articles were also searched by the two authors independently.

### Data Collection and Analysis

#### Data Extraction and Management

Two reviewers (DXW and ZYY) independently conducted the search according to the pre-designed inclusion and exclusion criteria. Necessary data and information from included studies were extracted by one reviewer and checked by the other. Discrepancies were jointly resolved by the two members.

#### Data Analysis

We used Review Manager 5.3 for the analysis of the extracted data. Along with 95% confidence interval (95%CI), the dichotomous outcomes were analyzed by relative ratio (RR) and the continuous outcomes by mean difference (MD). We evaluated heterogeneity by Chi-square test and *I*^2^ statistic calculation. We formulated our cut-off level at 50% for *I*^2^. When *I*^2^ > 50% or *P* < 0.05, indicating high heterogeneity among studies, the random-effects model was employed for meta-analysis. Otherwise, we chose the fixed-effects model since low heterogeneity was confirmed (18). We calculated the standard deviation for continuous outcomes as suggested by Cochrane (18). Study results were displayed through forest plots. Additionally, *P* < 0.05 indicated that the difference was statistically significant.

#### Assessment of Risk of Bias in Included Studies

Using a 12-category assessment of risk of bias, the quality of each eligible study was rated by two reviewers (DXW and ZYY) independently and defined as high, low, or unclear risk of bias. The criteria were recommended by Cochrane Back Review Group (19). Studies with an overall low risk of bias in six or more dimensions were classified as high-quality studies. Publication bias was estimated by funnel plot. Disagreements were resolved following discussion among the reviewers.

## Results

### Description of Studies

**Figure 1:**
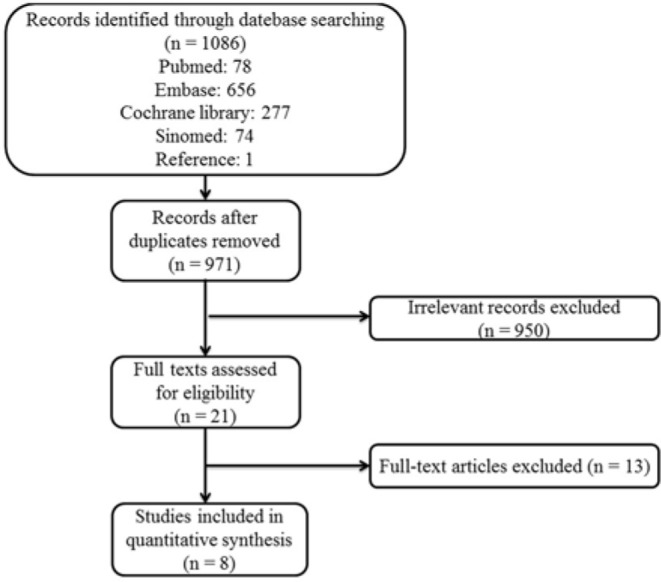
Study selection flow chart: Flow chart of the literature selection process for studies enrolled in this systematic review and meta-analysis. POTS, postural tachycardia syndrome.

A total of 1,086 original articles were identified initially from Cochrane, EMBASE, Pubmed and Sinomed databases. After removing duplicated studies, screening titles and abstracts as well as reviewing the full texts, 1078 articles were excluded and eight articles were accepted in our final analysis including four RCTs and four Non-RCTs. The flow chart of study selection is summarized in Figure 1.

The general characteristics of included studies are presented in Table 1. Of these eight trials, seven (10, 12–17) were reported in Chinese and one (11) in English. All of the studies focused on children and adolescents at an average age of 9.2–13.2 years. The β-blockers used in the selected studies were metoprolol, although in various dosages−0.5 mg/(kg·d) in two publications (11, 12), 12.5 mg/d in one (10) and 1.0 mg/(kg·d) for the others. The studies included shared similar intervention duration between 3 and 6 months. In terms of efficacy evaluation, seven studies referred to the WSS (20). Treatments that resulted in a reduction of 2-points or above in symptom score [four studies (13, 15–17)] or decrease in symptom score by 50 percent or above [three studies (10, 11, 14)] were defined, respectively, as effective. Only in one study (12) the treatment efficacy was evaluated by measuring the reduction of syncope frequency.

**Table 1:** Characteristics of included studies.

**Reference**	**Trial design**	**Participants (n)**			**Age (y), Mean ± SD**	**Treatment**		**Outcoms**	**Symptom tool**	**Efficacy**
		**Total**	**β-blockers**	**Control**		**β-blockers**	**Control**			
Chen et al. (12)	RCT	54	32	22	12.0 ± 2.6	Metoprolol 0.5 mg/(kg·d) bid; 3-6 months	ORS	-Effective rate	Improvement: Syncope decreased ≥50%	84.4 vs 40.9%
Chen et al. (11)	RCT	53	19	15	12.2 ± 2.4	Metoprolol 0.5 mg/(kg·d) bid; 3-6 months	Conventional therapy	-Symptom score -Blood pressure -ΔHR -Effective rate	Improvement: Symptom score decreased ≥50%	57.9 vs 53.3%
Lin et al. (10)	Non-RCT	192	54	54	11.4 ± 2.5	Metoprolol 12.5 mg/d bid; 3 months	ORS 500 ml	-Symptom score -Blood pressure -ΔHR -Effective rate	Improvement: Symptom score decreased ≥50%	72.2 vs 48.0%
Liu et al. (13)	RCT	21	14	7	9.24 ± 3.76	Metoprolol 1.0 mg/(kg·d) bid; 3 months	Oryzanol 10 mg/d tid	-Symptom score -Blood pressure -ΔHR -Effective rate	Improvement: Symptom score decreased by 2 points	85.7 vs 28.6%
Sun et al. (14)	RCT	92	34	26	13.2 ± 2.2	Metoprolol 1.0 mg/(kg·d) bid; 2 months	NS 250 ml bid	-Symptom score -Blood pressure -ΔHR -Effective rate	Improvement: Symptom score decreased ≥50%	94.1 vs 38.5%
Yang et al. (15)	Non-RCT	244	66	75	11.6 ± 2.5	Metoprolol 1.0 mg/(kg·d) bid; 3 months	ORS 500 ml	-Symptom score -Blood pressure -ΔHR -Effective rate	Improvement: Symptom score decreased by 2 points	80.3 vs 72.0%
Zhang et al. (16)	Non-RCT	30	20	10	13 ± 2	Metoprolol 1.0 mg/(kg·d) bid; 3 months	NS 250 ml bid	-Symptom score -Blood pressure -ΔHR -Effective rate	Improvement: Symptom score decreased by 2 points	80.0 vs 40.0%
Zhang et al. (17)	Non-RCT	118	10	39	11.4 ± 2.6	Metoprolol 1.0 mg/(kg·d) bid; 3 months	ORS 500 ml	-Symptom score -Blood pressure -ΔHR -Effective rate	Improvement: Symptom score decreased by 2 points	80.0 vs 74.4%

*RCT, randomized controlled trial; Non-RCT, non-randomized controlled trial; SD, standard deviation; ORS, oral rehydration salts; NS, normal saline; HR, heart rate; bid, twice a day*.

### Risk of Bias in Included Studies

**Figure 2:**
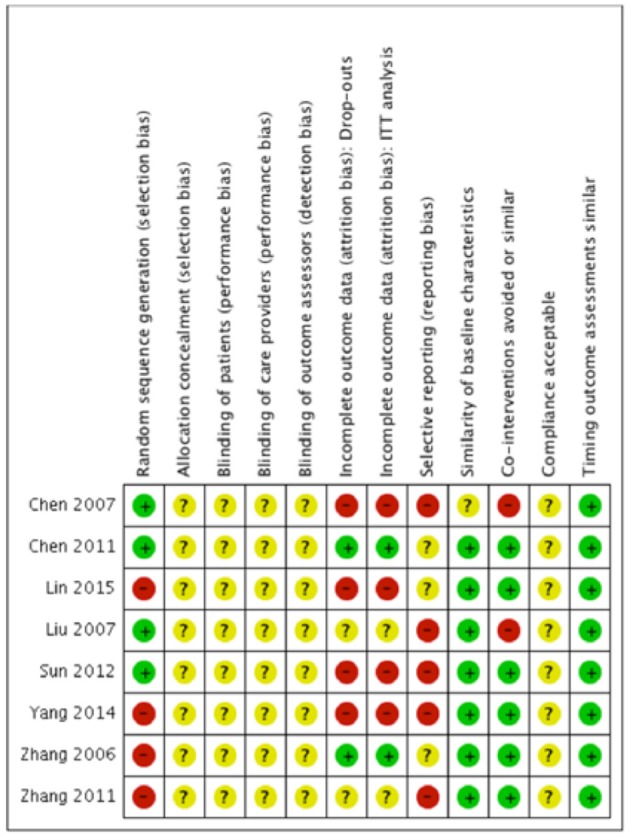
Risk of bias summary: Review author's judgments about each risk of bias item for each included study. “+” indicates certain criterion has been met and therefore suggests a low risk of bias; “–” indicates certain criterion has not been met and therefore suggests a high risk of bias; “?” indicates unclear risk of bias for lack of relative information.

**Figure 3:**
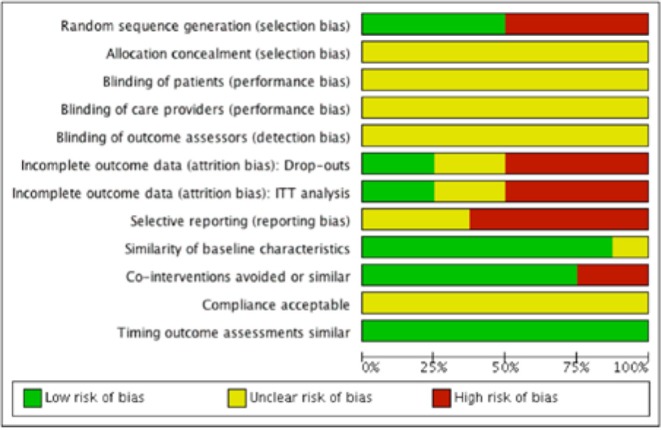
Risk of bias graph: Review author's judgments about each risk of bias item presented as percentages across all included studies.

We evaluated risk of bias in all enrolled studies using the criteria suggested by Cochrane Back Review Group. Because of unreported details, the risks of bias of most studies were defined as unclear. There is high risk of selection bias in four studies (10, 15–17) on account of non-randomized sequence generation. Four studies (10, 12, 14, 15) were decided as high risks of attrition bias due to incomplete outcomes. Five studies (12–15, 17) that failed to present all the pre-determined primary and secondary outcomes were considered as high risks of reporting bias. The study by Chen et al. (11) was the only study with low risk of bias in six categories of our bias assessment system, and therefore determined as overall high-quality. Our risk of bias estimation is summarized in Figures 2, 3.

#### Allocation

Of the four studies (11–14) reporting a random sequence generation, only Chen et al. (11) described the randomization process in detail. The other four studies (10, 15–17) that failed to mention allocation concealment were considered as high risk of bias under allocation category.

#### Blinding

None of the included studies stated a blinding process of participants, personnel, or outcome assessors.

#### Incomplete Outcome Data

There was no missing outcome data in two studies (11, 16). Four studies (10, 12, 14, 15) suggested a high risk of drop outs in outcome data and a lack of information for the intention to treat analysis. The risk of bias of the rest two studies (13, 17) was unclear due to no explicit statements in some of the outcomes regarding the number of participants.

#### Selective Reporting

Five studies (1, 12–15) that failed to report secondary outcomes such as the symptom score or the change of the heart rate were defined as high risks of selective reporting bias. The risk of bias assessment of the other three studies was classified as unclear due to unavailable study protocols.

#### Publication Bias

**Figure 4:**
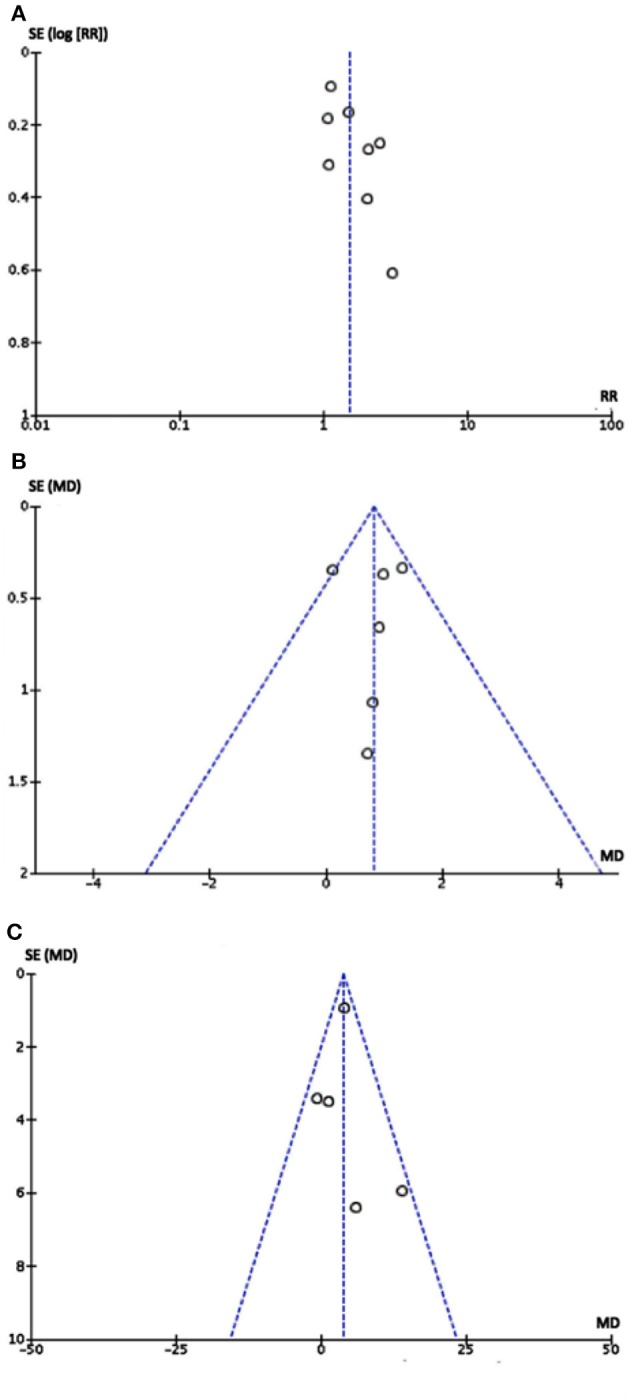
Funnel plot of eight studies: Each dot stood for one study. The distance between each dot and the upright line indicated the bias in each study. **(A)** Funnel plot of comparison between metoprolol with controls in therapeutic effect. **(B)** Funnel plot of comparison between metoprolol with controls in Δ symptom score. **(C)** Funnel plot of comparison between metoprolol with controls in Δ heart rate difference. As the funnel plots of three outcomes were visually symmetric implying that publication bias may not exist.

No publication bias was detected from the funnel plot (Figure 4) of primary and secondary outcomes visually, implying that the publication bias might not exist among the included studies.

#### Other Potential Sources of Bias

Baseline characteristics are similar between groups on demographic factors and important hemodynamic data in all studies except for the study by Chen et al. (12) which did not report this. As for “Co-intervention,” metoprolol was compared to conventional therapies including ORS (10, 12, 15, 17), oral normal saline (11, 14, 16) and oryzanol (13). In one study (12), metoprolol was used alone in the experimental group without parallel treatment as its control. Timing of outcome assessments was similar in all studies. Performance bias could not be assessed for that no descriptions of patient compliance could be found in any of the included studies.

### Outcomes

#### Primary Outcomes

**Figure 5:**
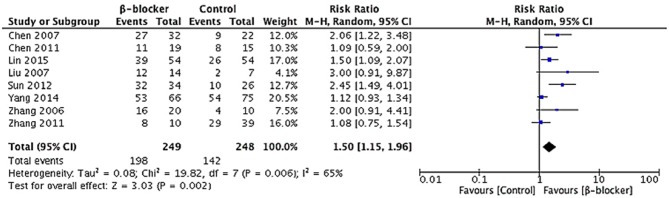
Forest plot of eight studies in efficacy rate for metoprolol vs. comparator: Heterogeneity analysis showed statistical heterogeneity among the studies (*p* < 0.05). A random-effects model was conducted. The analysis of total effects presented in the bottom. Risk ratio of efficacy rate analyzed by Mantel-Haensze test was summarized on the right. Each little square represented the RR value of each study along with a transverse line representing 95% CI. The rhombus below stood for the overall result of meta-analysis.

In respect of effective rate, data were available for all the studies. A random-effects model was conducted for meta-analysis. The Chi^2^ value for heterogeneity test of the risk ratio (RR) was 19.82 (*P* = 0.006) and *I*^2^ statistic 65%, which suggests statistical heterogeneity across studies. The studies enrolled reported 497 cases of pediatric POTS with 340 children improved after treatment, including 198 of the metoprolol group and 142 of the control group. The effective rate at the end of short-term follow-up in the metoprolol group was significantly higher than that of the control group (79.5 vs. 57.3%, RR = 1.50, 95% CI: 1.15–1.96, *P* = 0.002; Figure 5).

#### Secondary Outcomes

##### Δ Heart Rate Difference

**Figure 6:**
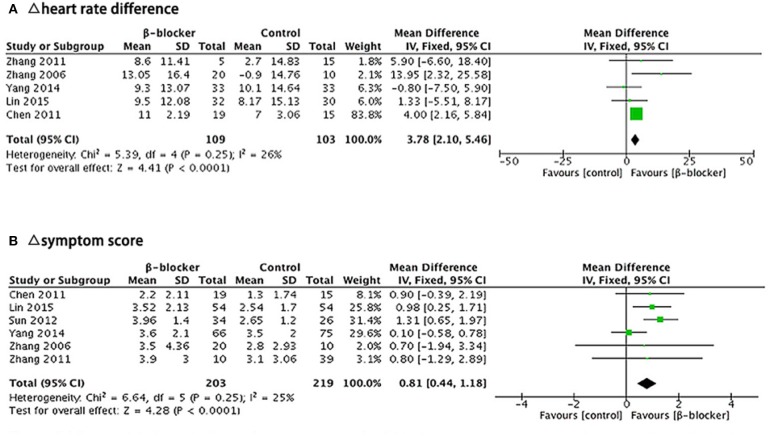
Forest plot for sub-group analysis: **(A)** Five studies, comparing Δ heart rate difference between β-blockers and comparator. **(B)** Six studies, comparing Δ symptom score for two groups. The analysis of total effects were presented in the bottom. Mean difference analyzed by inverse variance method was summarized on the right. Each little square represented the mean difference of each study along with a transverse line representing 95% CI. The rhombus below stood for the overall result of meta-analysis.

There were five articles (10, 11, 15–17) describing heart rate difference during standing test. We calculated the decrement in heart rate different (Δ heart rate difference) in each trial with fixed-effects model to analyze the results. Heterogeneity analysis of the subgroup showed a low level of heterogeneity (Chi^2^ = 5.39, *P* = 0.25, *I*^2^ = 26%). After the treatment, there was a reduction in the heart rate difference during standing test in both groups, but the heart rate change of the metoprolol group was significantly greater than the control group (MD = 3.78, 95% CI: 2.10–5.46, *P* < 0.0001; Figure 6A).

##### Δ Symptom Score

There were six articles (10, 11, 14–17) describing the symptom score outcome before and after treatment. We calculated the decrement in symptom score (Δ symptom score) in each trial and analyzed the results using a fixed-effects model. Heterogeneity analysis of the subgroup showed a low level of heterogeneity (Chi^2^ = 6.64, *P* = 0.25, *I*^2^ = 25%). The symptom score after treatment in both groups was lower than that before treatment, but the reduction of the symptom score was significantly greater in the metoprolol group than that of the control group (MD = 0.81, 95% CI: 0.44–1.18, *P* < 0.0001; Figure 6B).

##### Adverse Events

There was one article (14) missing the description of adverse events. Others had a record of that. Three studies (11, 13, 16) did not appear to report any side effects during the drug treatment. Chen et al. (12) showed that bradycardia occurred in three children without subjective symptoms. Lin et al. (10) reported that one child presented with fatigue and chest tightness. Yang et al. (15) discovered that three children complained of abdominal pain and one patient presented with decreased blood pressure. Zhang et al. (17) discovered that two children had stomach discomfort. None of the aforementioned adverse events were severe and all of the children went through full course of treatment.

## Discussion

In this systematic review and meta-analysis, we included eight studies that assessed β-blockers efficacy in treating POTS children and adolescents. All the eight trials collecting data on the effective rate of β-blockers showed that the efficacy of β-blockers were significantly higher than those of their comparable control treatments (79.5 vs. 57.3%, RR = 1.50, 95% CI: 1.15–1.96, *P* = 0.002), mainly with ORS or normal saline. In addition to this, β-blockers might also be more effective than controlled treatments in lowering the heart rate increment during standing test (MD = 3.78, 95% CI: 2.10–5.46, *P* < 0.0001). Finally, the decrement in symptom score is significantly greater than controlled treatments in the β-blocker group (MD = 0.81, 95% CI: 0.44–1.18, *P* < 0.0001). There was no reporting of severe adverse events that led to treatment discontinuation. However, the influence of the relatively small sample sizes and short follow-up period in most enrolled studies should not be neglected. All in all, we concluded that β-blockers are effective in treating POTS in children and adolescents, alleviating orthostatic intolerance, and improving hemodynamic abnormalities.

Postural tachycardia is the main hemodynamic feature of POTS children. When moving from a supine to a standing position, a healthy man would have blood pooling in the lower limbs due to the law of gravity, which is not perceivable thanks to the delicate regulatory mechanism culminating in a crucial increment of heart rate of 10–15 bpm. Unfortunately, owing to complex factors such as hypovolemia, autonomic dysfunction and neurohumoral dysregulation, POTS children often have a hard time making this normal adjustment through cardiac output compensation, resulting in a marked rise in heart rate, symptoms of orthostatic intolerance, and even cerebral hypoperfusion (21, 22).

Based on current understanding of potential etiology, several non-pharmacological treatments have been introduced into POTS treatment, such as reducing venous pooling by wearing lower-body compression garments or practicing resistance training for the thighs (23). Apart from that, studies have shown that 70% of POTS children have decreased intravascular volume, whose symptoms of orthostatic intolerance could be attenuated by increasing consumption of water and salt (24). In our review, we also found that ORS treatment was proved effective in most control groups. There is, still, a noticeable amount of POTS children who are not responding to the classic non-pharmacological ORS treatment (16), indicating the existence of other hemodynamic factors contributing to the clinical presentation of tachycardia.

The role of hyper-adrenergic state in the development of POTS has gained more and more attention in recent years. Zhang et al. (25) discovered that norepinephrine in some POTS patients increased significantly, the level of which was positively correlated with the severity of clinical presentation. Earlier studies reported that mutation of norepinephrine transporter (NET) might be one of the reasons for the elevated norepinephrine level (26). Other than that, the clearance mechanism of norepinephrine in POTS patients is damaged and their sympathetic activation is prominent (27, 28).

β-blockers were introduced into clinical practice based on the reasons listed above, the efficacy of which has been recognized to a certain extent, mainly through clinical observations and application experience. Under this context, we performed an updated review of the available evidence. Among the 249 POTS children treated with metoprolol, 198 cases reported symptom improvement. The pooled efficacy of metoprolol is 79.5%, which is significantly greater than the control group (57.3%), indicating that β-blocker is an effective way to treat POTS in children. The efficacy of β-blockers implies symptoms improvement as well as tachycardia alleviation.

There are two possible reasons supporting the efficacy of β-blockers. On one side, they could block cardiac β1 receptors, thus serving a negative inotropic effect. On the other side, they are capable of inhibiting renin secretion through the inhibition of β1 receptors of juxtaglomerular cells, resulting in a lowered norepinephrine level. Then, autonomic activity was reduced, followed by a decreased heart rate and improved orthostatic tolerance (29).

Although the theoretical basis for the efficacy of β-blockers seems rather solid, there are inconsistent results among the studies. The reasons might be multi-faceted. First of all, POTS is a heterogeneous disorder with complicated nosogenesis. Some researchers made the distinction between partial dysautonomic POTS and hyperadrenergic POTS, while others preferred the division of three subtypes (7, 30–32). Different pathogenesis among POTS subtypes indicates that individual patient may have different response to the same treatment. It has been reported that the plasma level of norepinephrine might serve as an efficacy predictor of metoprolol therapy for POTS in children and adolescents (25). None of the included studies described the baseline plasma norepinephrine level of POTS children, which might partly account for the individual difference in response to β-blockers. Secondly, the discrepancy between POTS diagnostic standards and the diversity of efficacy evaluation methods may contribute to the inconsistent results. Except for the study by Lin et al. (10) in which the latest diagnostic criteria of POTS (6) (an increment of heart rate of over 40 bpm within the first 10 min of HUT) were adopted, other seven studies uniformly determined a value of over 30 bpm heart rate elevation as standard, which could lead to heterogeneity in baseline hemodynamic level. As for efficacy assessment, all of the studies except for that by Chen et al. (12) used symptom score for the evaluation, but different standards were adopted when defining the key word “effective.”

Finally, the general limitation of trial design cannot be neglected. All studies included score of their targeted patients according to the WSS, which is a scoring system that requires self-evaluation of various symptoms at different time points. The WSS scale's relatively strong subjectivity and its generalization of symptoms of different severity could cripple the accuracy of the calculated efficacy that were held as the primary outcome for all studies. It is indeed worth pondering whether this WSS could evaluate the severity of POTS symptoms and efficacy of certain drugs both comprehensively and objectively.

Two systematic reviews and meta-analysis were published previously (33, 34). However, our present study showed that β-blocker was an effective therapeutic option for the treatment of POTS in children. With respect to the study inclusion criteria, we excluded the studies (35) that were ambiguous about the efficacy evaluation standard, while adding the studies (10) with larger sample-sized and more rigorous trial design. As for the pooled outcome, apart from the therapeutic efficacy that was adopted by both aforementioned reviews, we took on two new outcome assessment indexes—the “symptom score” and the “heart rate difference” in the evaluation.

In addition to the promising efficacy of metoprolol, its tolerance and safety seems rather acceptable. In our study, the rate of drug-related adverse effects in the metoprolol group was 4.0% (10/249), including abdominal discomfort, bradycardia, decreased blood pressure, fatigue and chest tightness. Although the present dosage of metoprolol, which is about 0.5–1 mg per kg everyday, presented a rare occurrence of unexpected events, a higher dose might be less well tolerated (8). Larger studies of longer follow-up period would be further required to identify rare or late-occurred adverse events.

Our review has several limitations that must be acknowledged. First of all, only articles written in English or Chinese are included in our study, leading to the inappropriate exclusion of trials published in other languages. Secondly, of the eight studies included, there are only four RCTs and the number of the studies with multi-center design and number of included sample size are not large enough. There are selective reporting with respect to symptom score in two studies and hemodynamic changes in three studies. Four studies lacked long-term follow-up, and the description of blinding process and patient compliance was unavailable in most articles. All of the above might affect the result.

At present, β-blockers have been used to in treating POTS children in many studies, but it is unclear whether there are any significant differences in its therapeutic efficacy over age. Convincing evidence derived from large scale RCTs that supports its efficacy is still a vacancy (36, 37). Through this systematic review and meta-analysis, we concluded that β-blocker was effective in treating POTS in children and adolescents, alleviating orthostatic intolerance, and improving hemodynamic abnormalities. However, limited by the disease's elusive pathogenesis, baseline difference of patients and the overall deficiency in study design, more studies of RCT and/or multicenter-based clinical studies are still in need before reaching a solid consensus. As for the research direction, we recommend that more efforts should be made for the establishment of a uniform standard for efficacy assessment, and also for the exploration of potential connections between symptoms and their underlying mechanisms, in order to offer reliable basis for a more evidence-based management of this complex disorder.

## Conclusion

β-blockers are effective in treating POTS in children and adolescents, alleviating orthostatic intolerance, and improving hemodynamic abnormalities.

## Author Contributions

XD and YZ conceived the study, performed the data extraction, and coordinated data collection and analysis. Initial manuscript was drafted by XD and YZ collectively. YL and JD reviewed and critically revised the drafts of the manuscript and approved the final version as submitted.

### Conflict of Interest

The authors declare that the research was conducted in the absence of any commercial or financial relationships that could be construed as a potential conflict of interest.
